# Vismodegib treatment in advanced basal cell carcinomas: Real‐life experience

**DOI:** 10.1111/dth.15195

**Published:** 2021-11-16

**Authors:** Alessia Villani, Gabriella Fabbrocini, Claudia Costa, Massimiliano Scalvenzi

**Affiliations:** ^1^ Dermatology Unit, Department of Clinical Medicine and Surgery University of Naples Federico II Naples Italy

Dear Editor

1

It is well‐known that the hedgehog‐signaling pathway plays a central role in basal cell carcinoma (BCC) pathogenesis; vismodegib and sonidegib are two oral sonic hedgehog signaling pathway inhibitors approved for the treatment of advanced BCCs that are not eligible for surgery or radiotherapy. They connect to the smoothened protein, thus deactivating the hedgehog pathway and resulting in tumor growth inhibition.^1^, ^2^ Prospective trials and real‐life experiences have demonstrated the efficacy and safety of vismodegib in treating advanced BCCs.^3^ We read with great interest the article recently written by Gurbuz et al.^4^, ^5^, ^6^ exploring real‐life data on vismodegib efficacy and tolerability in locally advanced (la)BCCs and we also want to report the experience of our center. Data of patients with BCC presenting to the University of Federico II Naples were retrospectively reviewed; all patients with laBCC or multiple primary BCCs treated with vismodegib were included in the study. Patients were treated with 150 mg of oral vismodegib per day. Age, sex, and tumor location were recorded. Patients' data are reported in Table 1. Moreover, all the adverse events related to the drug were recorded during the monthly follow‐up visit. Response status of patients was evaluated after 6‐month treatment and divided in: complete remission (CR; >80% of tumor regression), partial remission (PR; from 50% to 80% of tumor regression), stable disease (SD; from 10% to 50%) and no response (NR; <10%). Forty‐eight patients (35 males and 13 females) with a median age of 74.5 years (range: 43–95) were included. The 83% (*n* = 40) of patients had one laBCC whereas the 17% (*n* = 8) presented with multiple primary BCCs. BCCs were located on head and neck in 33 (82.5%) patients, trunk in 3 (7.5%) patients and on the upper and lower limbs in 4 (10%) patients. Medium duration of vismodegib treatment was 6 months (ranging from 1 to 15 months). Five patients (10.4%) discontinued treatment due to no‐compliance after 2 months, one patient after 5 months being non‐responder, and one patient after 2 months for adverse effects. Regarding therapeutic response after six‐month treatment with vismodegib: 16 patients (33.3%) reported CR, 15 patients (31.2%) showed PR and 10 patients (20.9%) SD. The 14.6% (*n* = 7) were non‐responders to treatment. The longest follow‐up period after vismodegib discontinuation was 22 months, with only two recurrences of the disease. Although 40 patients (84%) experienced more than one adverse event, vismodegib showed to be a well‐tolerated drug; muscle spasms, dysgeusia, and alopecia were the adverse events most frequently described with a percentage of 77%, 79%, and 58%, respectively. The majority of patients reported a mild to moderate grade (grade 1 or 2) according to National Cancer Institute Common Terminology Criteria for Adverse Events (CTCAE version 5). Vismodegib is the first hedgehog pathway inhibitor approved for the treatment of laBCC and metastatic BCC. As reported in previous studies,^5^, ^7^ in our retrospective analysis, vismodegib treatment was effective and well‐tolerated. Limitations of the study were the retrospective design and the small sample size. Real‐life data on larger cohort of patients are still required.

**TABLE 1:** Data regarding patients treated with vismodegib

Patients' characteristics	
Median age	74.5 years (range: 43–95)
Male:Female (*n*;%:*n*;%)	(35; 72.9%): (13; 27.1%)
Mean number of BCC per patient at baseline	2.2 per patient
Mean BCC diameter at baseline	5.3 cm
Tumor location	Head–neck: 33 (82.5%) Trunk: 3 (7.5%) Upper and lower limbs: 4 (10%)
Previous treatment	Surgery (25: 52%) Cryotherapy (3; 6.2%) Topical treatment (3; 6.2%) Radiotherapy (2; 4.1%) No treatment (13; 31.5%)

## CONFLICT OF INTEREST

The authors declare no potential conflict of interest.

## AUTHOR CONTRIBUTIONS

*Idea and design*: Alessia Villani, Gabriella Fabbrocini, and Massimiliano Scalvenzi. *Data collection*: Claudia Costa. *Revision of the article*: Alessia Villani and Massimiliano Scalvenzi.

## ETHICS STATEMENT

Approval of ethical committee was obtained from the University Federico II of Naples, Italy.

## Data Availability

The data that support the findings of this study are available on request from the corresponding author. The data are not publicly available due to privacy or ethical restrictions.
